# Supplementary material for: Associated factors of prosthetic rehabilitation in specialized dental care in Brazil: a cross-sectional study
Source: BMC Res Notes. 2023 Apr 17;16:55. doi: 10.1186/s13104-023-06318-x (PMC10111834; doi:10.1186/s13104-023-06318-x)
Supplement: Supplementary file 1 — Supplementary Material 1 [file 13104_2023_6318_MOESM1_ESM.docx]

**Associated factors of prosthetic rehabilitation in specialized dental care in Brazil: a cross-sectional study**

Inara Pereira da Cunha^1^

Valéria Rodrigues de Lacerda^2^

Manoelito Ferreira Silva Junior^3^

Rafael Aiello Bomfim^4^

^1^Public Health School, Campo Grande, Brazil. E -mail: [inara-pereira@hotmail.com](mailto:inara-pereira@hotmail.com) ORCID: https://orcid.org/0000-0002-5330-6869

^2^Federal University of Mato Grosso do Sul, Campo Grande, Brazil. E-mail: [val99@terra.com.br](mailto:val99@terra.com.br) ORCID: https://orcid.org/0000-0001-6901-0520

^3^State University of Ponta Grossa, Ponta Grossa, Paraná, Brazil. E-mail: [manoelito_fsjunior@hotmail.com](mailto:manoelito_fsjunior@hotmail.com) ORCID: https://orcid.org/0000-0001-8837-5912

^4^Federal University of Mato Grosso do Sul, Campo Grande, Brazil. E-mail: [aiello.rafael@gmail.com](mailto:aiello.rafael@gmail.com) ORCID: <https://orcid.org/0000-0002-6478-8664>

**Corresponding author**

Inara Pereira da Cunha

Public Health School

Av. Sen. Filinto Müler, 1480 - Pioneiros, Campo Grande - MS, 79074-460

E-mail: [inara-pereira@hotmail.com](mailto:inara-pereira@hotmail.com)

Phone: +55 (67) 3345-8000

**Abstract**

Objectives: This study aimed to analyze the individual and contextual factors associated with prosthetic rehabilitation in Dental Specialty Centers (DSC) in Brazil. Methods: A cross-sectional study, with secondary data from modules II and III of the External Assessment of the 2nd Cycle of the National Program for the Improvement of Access and Quality (PMAQ) of DSCs, was conducted in 2018. Individual variables considered were socioeconomic conditions and perceptions about the structure and service of the DSC. Contextual variables were related to DSC. We considered the region of the country (capital or countryside), geographic location and work process of the DSC for prosthetic rehabilitation. The association between individual and contextual variables and prosthetic rehabilitation in the DSC was analyzed by multilevel logistic regression. Results: Ten thousand three hundred ninety-one users from 1,042 DSC participated. Of these, 24.4% used dental prosthesis and 26.0% performed at the DSC. In the final analysis, performed dental prostheses in the DSC individuals with less education (OR=1.23; CI95%:1.01-1.50) and residents of the same city as the DSC (OR=1.69; CI95%:1.07-2.66). Moreover, at a contextual level, DSCs of the countryside (OR=1.41; CI95%:1.01-1.97) were associated with the outcome. Conclusion: Individual and contextual factors were associated with prosthetic rehabilitation in the DSC.

**Keywords:** Dental Prosthesis; Unified Health System; Oral health Services; Cross-sectional Studies.

**Introduction**

Oral health conditions are a challenge for public health.^1^ Until the year 2017, approximately 3.5 billion people in the world had oral problems, and 267 million were people who had tooth loss.^1^ In Brazil, the last epidemiological survey of oral health  showed an increase, in tooth loss with age. The decayed, missing and filled teeth (DMF-T) rate represented 5.8% of the rate among young people, 44.7% among adults and 92% among the elderly.^3^

Tooth loss compromises the functionality of the dentition, leading to difficulties in speech and the act of smiling^4,5^ and, consequently, affecting individuals' social and emotional interactions.^6^

Thus, prosthetic rehabilitation is necessary since the replacement of teeth lost by prostheses restores chewing, promotes better nutrition, and provides well-being and facial aesthetics, which leads to increased quality of life.^7^

The prosthetic rehabilitation offered by the Unified Health System (SUS) is provided by the National Oral Health Policy (PNSB) through the "Brasil Sorridente" program. The laboratory manufacture of dental prostheses, which include removable mandibular partial dentures, removable maxillary partial dentures, total mandibular dentures, total maxillary dentures, and fixed/adhesive coronary/intra-articular prostheses, are the responsibility of the regional dental prosthesis laboratories (LRPD) or private laboratories hired by the management of the services, as provided for in Ordinance No. 1,825 of 2012. The clinical phase of dental prostheses, including impressions, cementation, adaptation, and guidance to users regarding use, hygiene, and post-use adjustment, is carried out by dentists working in Primary Health Care (PHC) or by prosthetics from the Dental Specialty Centers (DSC).

The DSCs were created to increase the population's access to specialized procedures, continuing the care initiated by the PHC according to referral protocols established by the services.^8,9^ Despite efforts to resolve a greater supply of prostheses in the SUS, in 2014, approximately 22,653 complete dentures and 10,070 removable partial dentures were delivered per month in the country10, an insufficient number to meet the dental prosthesis needs of the 9,501,160 Brazilians aged between 65 and 74 years.

Furthermore, the uneven distribution of public oral health facilities stands out10. Of the 780 DSCs in the Brazilian regions in 2014, 325 had established working processes with the LRPD. Considering the proportion of capitals with DSCs, the North and Northeast regions were the least favored.^10^ Thus, in addition to the limited and uneven supply of dental prostheses in DSCs, other organizational and individual factors of SUS users may affect access to rehabilitation treatment.^11^ Thus, it is necessary to investigate the factors that permeate the prosthetic rehabilitation of users in specialized dental services to collaborate with the work process at this level of oral health care.

This study aimed to analyze the individual and contextual factors associated with prosthetic rehabilitation in Dental Specialty Centers in Brazil.

**Main Text**

**Methods**

This is a cross-sectional study with data extracted from the database on the second cycle of External Evaluation of the National Program for Improving Access and Quality of Dental Specialty Centers (PMAQ-CEO – in Portuguese), available on the website of the Secretary of Primary Health Care, from the Ministry of Health (https://aps.saude.gov.br/ape/pmaq/ciclo2ceo/).

The second cycle of the PMAQ-CEO was carried out in Brazil in 2018 and presented three stages of development. The last was the on-site verification of the quality standards established by the program (external evaluation). In this stage, a trained external evaluator, independent of the service, applied a questionnaire divided into three modules. Module I related to evaluating the structure, equipment, instruments, and supplies of the facility. Module II, which included data related to the work process, the organization of the service and the care of the users, was answered by the managers of the DSC and a dentist of any specialty. Module III, designed to collect data on user satisfaction and perceptions of specialized oral health services regarding access and use, was administered to users at the DSC. Detailed information about the program is available in the PMAQ-CEO 2nd Cycle Instruction Manual.^12^

The present study extracted data from modules II and III of the PMAQ-CEO. The spreadsheets exported to the Microsoft Office Excel 2010 program were merged using the National Registry of Establishment number as a common identifier, as reported in a previous study.^13^ In this way, linking the data provided by the DSC's managers and dentist to the users' data was possible.

The sample of users participating in the external evaluation of the 2nd Cycle of the PMAQ-CEO was of convenience. As inclusion criteria, only users aged 18 and over were considered for the interview. Those present for the first time in the DSC were excluded, as provided in the Ministry of Health's PMAQ-CEO external evaluation manual. Each field evaluator was trained to apply the instrument to 10 users aged 18 and over who were present at the DSC on the day of the external evaluation.^12^

The dependent variable was extracted from module III, based on the following question: “Where did you get your dental prosthesis?”. The response options were operationalized for analysis purposes in: “In the DSC (in this or another)” or “Other” (Primary Health Unit, Private Clinic or Private Practice, Other).

The independent contextual variables were the region of the country (including all regions considered) and the location of the DSC (urban or rural). In addition to the questions from module II, namely: referral for prosthodontics impressions at the DSC (yes/no), waiting list management (yes/no) and presence of predefined places for referral of primary care users to clinical prosthodontist (yes/no/no service in this specialty), estimated waiting time for the user to be seen by the clinical prosthodontist at the DSC (dichotomized in: ≤2 months, >2 months, no information), number of people in the queue waiting to be seen for a prosthesis (dichotomized by the median into ≤123, >123 days and no information), suspension of DSC care in the past 12 months due to lack of supplies or instruments (yes/no), average number of dentures delivered per month (dichotomized into: ≤25 or >25).

Questions from module III were also considered as individual independent variables related to sociodemographic aspects, such as sex (male/female), age (categorized as <44 years old, from 45 to 64 years old or ≥65 years old), self-reported ethnicity/skin-color (yellow/indigenous, white, brown, black) marital status (single, married, divorced/widowed), retirement (yes/no), family income (≤1 minimum wage, > 1 minimum wage), family allowance benefits (yes/no), level of education (up to complete secondary education/at least complete secondary education).

In addition, it was considered whether the respondent lived in the same municipality as the DSC (yes/no), whether the home was covered/accompanied by the Family Health Strategy (yes/no), the mean time to reach the DSC (dichotomized by the mean ≤ 20 minutes and > 20 minutes), whether the opening hours met the needs (yes/no), reception when looking for the DSC (very nice, good, fair or poor, very fair), good conditions of use of the facilities of the DSC (yes/no), the general opinion of the service received from the DSC (very nice, good, fair or poor, very fair). The sample size was determined by the PMAQ-CEO Coordination in accordance with the program manuals.

Initially, frequency distribution tables were built. Then, analyses of the associations between prosthesis performance in a DSC with individual and contextual variables were performed. For this, simple and multiple multilevel logistic regression models were used. First, multilevel models were performed to consider possible dependencies between the observations of patients from the same DSC. The variables of the first level (individual) and the second level (contextual/DSC) were then considered in the model. Finally, using the empty model, using only the intercept, it was possible to calculate the intraclass correlation coefficient, estimating the proportion of the total variance due to the context (DSC).

In the multiple models, we selected the variables that presented p<0.20 in the crude analyses. Next, the first-level variables were included in the model, remaining with p≤0.05 after adjustments for the first-level variables. After the second-level variables were included, those with p≤0.05 remained in the final model after adjustments for the other variables. Finally, the crude Odds Ratio (OR) were calculated and adjusted to 95% confidence intervals (95%CI). The QIC evaluated the fit of the models. The analyzes were performed using the R and Statistical Analysis System (SAS) programs.

The study was approved by the Research Ethics Council under protocol 23458213.0.1001.5208, following resolution 466/2012 of the National Health Council. All participants received and signed the Free and Informed Consent Term in two signed copies.

**Results**

Of the 10,391 respondents, out of 1,042 DSCs in the country, 24.4% (95%CI: 23.6%; 25.2%) used dental prostheses. Of individuals who reported using dental prostheses, 26.0% performed in a DSC and the rest in another location.

Tables 1 and 2 present the frequency distributions of patients who used dental prostheses according to the independent variables. Most of the sample was female (68.3%), aged between 45 and 64 years (57.0%), married (54.1%), not retired (65.1%), with a family income of up to one minimum wage (55.6%) and a level of education up to incomplete high school (67.2%). In addition, 93.2% live in the municipality of DSC, with Family Health Strategy (FHS) coverage (81.9%). It was observed that 97.2% and 97.1% found the reception and service provided by the DSC to be good, respectively, but for 96.7%, the DSC's opening hours do not meet their needs. It was also possible to identify that the majority were patients of DSCs from the countryside (85.0%), with 72.0% patients of DSCs who perform dental prosthesis impressions and 75.0% of DSCs who manage the waiting list.

When the variables were adjusted (final model), it was observed that the prevalence of patients who had their prosthesis performed in a DSC was significantly higher among those with a lower level of education [OR=1.23 (95%CI 1.01;1 .50)], who live in the municipality where the DSC is located [OR=1.69 (95%CI 1.07;2.66)] and among those patients with DSC in the countryside [OR=1.41(95%CI 1 .01;1.97)] (Table 3).

**Discussion**

The present study evaluated the individual and contextual factors associated with prosthetic rehabilitation in DSC in Brazil. Still, it identified that the prevalence of users who had dental prostheses produced by the DCS is low. At an individual level, users with a lower level of education and who lived in the same city where the DSC is located, and at a contextual level, those who accessed the DSC in the countryside were more likely to have their prosthetic needs met.

There are financial, geographic and organizational barriers that compromise users' access to the services offered by the DSC.^14^ However, few investigations explore access to prosthetic rehabilitation promoted by this service. When it comes to barriers to accessing dental prostheses in the DSC, the findings revealed that people with a lower level of education were more likely to have a prosthesis in the DSC, a finding also verified in the literature.^15^ Although this seems optimistic, this data may reflect attention that only comes to individuals dependent on the SUS, in which the wait and realization occur only because they do not have another viable option for its execution. Thus, reducing the waiting list, in addition to making the service more resolute, can minimize the problems inherent to a population that, when not attended, has no other way of performing the dental prosthesis.

Obtaining a dental prosthesis was higher among users who lived in the same municipality as the DSC. Therefore, despite all efforts to facilitate the process of regionalization of services in an inter-municipal way, through referrals to specialties through the Consortium Agreed Programming (PPC) among the consortium entities,^16^ still end up privileging the reference municipality. In the PPC, the state and the countryside of a specific health region financially agree to maintain the DSC and its operation according to the existing human resources, establishing the number of vacancies for the countryside and state that compose it.^17^ In general, services use the Regulation System (SISREG) to manage referrals between Primary Health Care and the Regional Reference Center for Dental Specialty.^18,19^

However, in the first external evaluation of the PMAQ-CEO, carried out in 2014, it was observed that of the 876 DSCs distributed in Brazil, only 358 (38.5%) had clinical protocols for referring users of primary health care to the dental prosthesis specialty,^20^ which also demonstrates a low agreement for specialized dental prosthesis services in Brazil. It can also be understood that the larger population size of the municipality of reference for regional health makes the citizens of these municipalities more likely to be served in these establishments. Therefore, the relationship between user access and decentralization and regionalization of dental care services at a secondary level has not been established.^18^ This seems to depend on the distribution characteristics of the DSC, its coverage areas, transport logistics, protocols and work practices, and the demand profile for specialized care. It is suggested that other studies explore this theme, considering the specialty of dental prostheses. After all, strengthening the regionalization of services makes it possible to take specialized, more expensive technologies to the population of the municipalities associated with a region, optimizing resources and expanding the guarantee of oral health care.^21^

It was also observed that users living in rural areas were more likely to solve their prosthetic problems at the DSC than users living in urban areas of the capital. In general, the literature reports difficulties for the prosthodontist service to achieve good productivity.^9,22,23^ Some specialties offered by the DSC have better performance in terms of targets for procedures, which may be related to the sociodemographic conditions of the communities, such as the Human Development Index, gross domestic product, illiteracy rate, poverty and FHS coverage.^24^

Although one study showed non-compliance with oral surgery targets was associated with the larger population size of the cities studied,^25^ no studies with this evaluation were found for the prosthodontic specialty.

Finally, it is worth clarifying that the PNSB established prosthetic rehabilitation in the SUS in 2004. Through this policy, the Dental Prosthesis Laboratories (LRPD) were structured. However, by 2013, 1,465 LRPD were qualified, unequally distributed in the country, without considering epidemiological indicators and the population's prosthetic needs, with production below meeting the population's demands - rates of 15.81 total dentures delivered per month per 100,000 inhabitants.^26^ The low productivity and distribution of LRPD can affect the offers of the prosthesis specialty at the DSC, since these laboratories collaborate with the manufacture of prostheses requested both by the oral health teams of primary care and mobile dental teams units (UOM) and DSC.

According to the Department of Health's Strategic Management Support Room, 2,524,403 dentures were provided in secondary care between 2010 and 2015. Although many dentures have been delivered over the years, there is a deficit in access to prosthetic rehabilitation at this level of care. In this sense, the pace of expansion of services has not kept pace with the demand for services. It is noteworthy that the specialty of prosthetics is not included in the list of minimum specialties available to the DSC, and its inclusion may be a local management decision.

The expansion of access to dental prostheses in the SUS has been discussed,^28^ including encouraging the provision of dental prostheses in PHC. However, the analysis of the performance of 18,114 oral health teams inserted in the PHC in 2014 revealed that less than half of them (43%) delivered some type of dental prosthesis.^29^ Comparing the performance of the oral health teams in PHC, between the years 2011/2012 and 2013/2014, there was a 0.8% increase in taking impressions for prostheses, indicating a low number of teams in PHC that perform the procedures for the prosthetic rehabilitation of users.^30^

It is also essential to consider that the manufacture of dental prostheses is recommended through several stages and requires inputs, material resources, and technical skills from the dentist.^27^ Characteristics of the dental practices included in the DSC, and the structure of these establishments, need to be considered. It is expected that the largest number of deliveries of prostheses to the SUS user population is through this service, which is a hypothesis to be tested for better readjustment and reallocation of resources considering all access points of the oral health care network.

**Conclusion**

Individual factors such as education level and living in the same municipality as the DSC, and contextual factors such as accessing a DSC located in the countryside, are associated with prosthetic rehabilitation in the specialized dental care of the SUS.

**Limitation**

As a limitation, it is emphasized that the design of the PMAQ-CEO data collection considered the approach only to users present in the establishment, as informants by free adhesion, thus veiling the data and the perception of users not present. Thus, the convenience sample may not characterize the entire population assisted by dental specialties, so we cannot generalize our findings to the country.

**Authors' contributions**

Cunha IP, Lacerda VR, and Bomfim RA participated in the conception, design, and data collection. Silva Junior MF anda Cunha IP, participated in the article writing. Cunha IP, and Bomfim RA participated in the critical review. All Authors read and approved the manuscript.

**References**

1. Bernabe E, Mercenes W, Hernandez CR, Bailey J, Abreu LG, Alipour V, et al. Global, Regional, and National Levels and Trends in Burden of Oral Conditions from 1990 to 2017: A Systematic Analysis for the Global Burden of Disease 2017 Study. J Dent Res. 2020;99(4):362–73.

2. Peres MA, Barbato PR, Reis SCGB, Freitas CHS de M, Antunes JLF. Perdas dentárias no Brasil: análise da Pesquisa Nacional de Saúde Bucal 2010. Rev Saúde Pública. 2013;47(suppl 3):78–89.

3. Brasil. SB Brasil 2010: pesquisa Nacional de Saúde Bucal: resultados principais. 1a̲ edição. Brasília - DF: Ministério da Saúde : Secretaria de Atenção à Saúde : Secretaria de Vigilância em Saúde; 2012.

4. Nordenram G, Davidson T, Gynther G, Helgesson G, Hultin M, Jemt T, et al. Qualitative studies of patients' perceptions of loss of teeth, the edentulous state and prosthetic rehabilitation: A systematic review with meta-synthesis. Acta Odontologica Scandinavica. 2013;71(3–4):937–51.

5. Souza JGS, Costa Oliveira BE, Martins AMEDBL. Contextual and individual determinants of oral health-related quality of life in older Brazilians. Qual Life Res. 2017;26(5):1295–302.

6. Özhayat EB. Influence of self-esteem and negative affectivity on oral health-related quality of life in patients with partial tooth loss. Community Dent Oral Epidemiol. 2013; 41(5):466-72.

7. Ali Z, Baker SR, Shahrbaf S, Martin N, Vettore MV. Oral health-related quality of life after prosthodontic treatment for patients with partial edentulism: A systematic review and meta-analysis. J Prosthet Dent. 2019 Jan;121(1):59-68.e3.

8. Rios LRF, Colussi CF. Avaliação normativa dos Centros de Especialidades Odontológicas, Brasil, 2014. Saúde debate. 2019;43(120):122–36.

9. Rebelo MAB, Emmi DT, Herkrath FJ, Meira GF, Goes PSA, Vettore MV. Need of Dental Prosthesis in Older People and the Offer of Specialized Care in the Unified Health System, Brazil. Pesqui. Bras. Odontopediatria Clín. Integr. 2018;18(1):3201.

10. Colussi CF, Patel FS. Uso e Necessidade de Prótese Dentária no Brasil: avanços, perspectivas e desafios. Sau. & Transf. Soc. 2016;7(1):41-48.

11. Pinto V de PT, Teixeira AH, Santos PR, Araújo MWA de, Moreira MÁG, Saraiva SRM. Avaliação da acessibilidade ao Centro de Especialidades Odontológicas de abrangência macrorregional de Sobral, Ceará, Brasil. Ciênc saúde coletiva. 2014;19(7):2235–44.

12. Brasil. Ministério da Saúde. Secretaria de Atenção à Saúde. Departamento de Atenção Básica. Programa Nacional de Melhoria do Acesso e da Qualidade dos Centros de Especialidades Odontológicas (Pmaq-CEO): manual instrutivo 2º Ciclo (2015-2017) / Ministério da Saúde, Secretaria de Atenção à Saúde. Departamento de Atenção Básica. – Brasília: Ministério da Saúde, 2017.

13. Bomfim RA, da Cunha IP, Lacerda VR. Health ombudsman and racial inequities in Dental Specialities Centers performance in Brazil: A multilevel analysis. Community Dent Oral Epidemiol. 2022 Feb;50(1):11-18.

14. Machado FC de A, Silva JV, Ferreira MÂF. Factors related to the performance of Specialized Dental Care Centers. Ciênc saúde coletiva. 2015;20(4):1149–63.

15. Ladeia MMF, Lessa AMG, Lessa ÂG. Análise dos prontuários odontológicos do serviço de prótese total nos anos de 2016 e 2017 em uma cidade de médio porte da Bahia. Salusvita. 2019;38(1):14.

16. Brasil. Diretrizes para a programação pactuada e integrada da assistência à saúde. Brasília, Brazil: Ministério da Saúde, Secretaria de Atenção à Saúde, Departamento de Regulação, Avaliação e Controle de Sistemas; 2006. 150 p. (Série Pactos pela saúde).

17. Fonseca EP, Silva Junior JP da, Vedovello SAS, Souza LZ, Pereira AC, Meneghim M de C. Fatores associados às faltas em tratamentos ortodônticos em centro de especialidades odontológicas. Ciênc saúde coletiva. 2018;23(1):287–94.

18. Magalhães MBP de, Oliveira DV de, Lima RF de, Ferreira EF e, Martins R de C. Avaliação da atenção secundária em endodontia em um Centro de Especialidades Odontológicas (CEO). Ciênc saúde coletiva. 2019;24(12):4643–54.

19. Figueiredo N, de Goes PSA, Martelli PJ de L. Relatório do 1^o^ ciclo da avaliação externa do Programa de Melhoria do Acesso e da Qualidade dos Centros de Especialidades Odontológicas (AVE-PMAQ/CEO): resultados principais [Internet]. 2016 [citado 3 de setembro de 2021]. Disponível em: http://189.28.128.100/dab/docs/portaldab/publicacoes/relatorio_1_ciclo_pmaq_ceo.pdf

20. Silva Junior CL, Almeida PF de, Martins Filho MT, Lima LD de. Implementação dos Centros de Especialidades Odontológicas no contexto da regionalização da saúde no estado do Ceará, Brasil. Physis. 2020;30:e300404.

21. Moura FRR de, Tovo MF, Celeste RK. Cumprimento de metas dos Centros de Especialidades Odontológicas da Região Sul do Brasil. Rev salud pública. 2017;19(1):86–93.

22. Santana DA de, Santos LP de S, Carvalho FS de, Carvalho CAP de. Desempenho dos Centros de Especialidades Odontológicas de uma Região de Saúde da Bahia. Cad saúde colet. 2015;23:261–7.

23. Herkrath FJ, Gonçalves MJF. Desempenho dos Centros de Especialidades Odontológicas frente ao quadro sociodemográfico dos municípios do Amazonas, Brasil, 2009. Saúde Debate. 2013;37(96):11.

24. Thomaz EBAF, Sousa GMC de, Queiroz RO de S, Coimbra LC. Avaliação do cumprimento das metas de produtividade em Centros de Especialidades Odontológicas no Maranhão, 2011. Epidemiol. Serv. Saúde. 2016;25(4):807–18.

25. Aguiar VR, Celeste RK. Necessidade e alocação de laboratórios regionais de prótese dentária no Brasil: um estudo exploratório. Ciênc saúde coletiva. 2015;20:3121–8.

26. Brasil. Brasil Sorridente/Centro de Especialidades Odontológicas - CEO/Prótese Dentária - Conjuntos de dados - Portal Brasileiro de Dados Abertos [Internet]. 2020 [citado 4 de setembro de 2021]. Disponível em: https://dados.gov.br/dataset/mspainelsage_30

27. Corrêa HW, Bitencourt FV, Nogueira AV, Toassi RFC. Saúde bucal em usuários da atenção primária: análise qualitativa da autopercepção relacionada ao uso e necessidade de prótese dentária. Physis. 2016;26(2):503–24.

28. Cunha MAGM, Matta-Machado ATG, Lucas SD, Abreu MHNG. Availability of Dental Prosthesis Procedures in Brazilian Primary Health Care. Biomed Res Int. 2018;2018:4536707.

29. Abreu MHNG de, Morato A de LFN, Marinho AMCL, Cunha MAM, Mendes S da R. What Has Changed in the Dental Prosthesis Procedures in Primary Health Care In Brazil? Braz Dent J. 2019;30(5):519–22.

30. Cunha MA, Vettore MV, dos Santos TR, Matta-Machado AT, Lucas SD, Abreu MHNG. The Role of Organizational Factors and Human resources in the Provision of Dental Prosthesis in Primary Dental Care in Brazil. *Int. J. Environ. Res. Public Health.* 2020;17(5):1646.

**Table 1.** Frequency distribution of sample patients as a function of individual variables, PMAQ-CEO, Brazil, 2018.

| **Variables** | **n (%)^a^**  **(n= 2.706)** | |  | | **n (%)^b^ of patients**  **that made prosthesis in DSC (n= 703)** | |  |
| --- | --- | --- | --- | --- | --- | --- | --- |
| **The place where the prosthesis was made.** | |  | |  | |  | |
| DSC (or other) | 703 (26.0) | |  | | - | |  |
| Other places | 2,003 (74.0) | |  | | - | |  |
| **Gender** |  | |  | |  | |  |
| Male | 857 (31.7) | |  | | 210 (29.7) | |  |
| Female | 1,849 (68.3) | |  | | 493 (70.3) | |  |
| **Age (years)^c^** |  | |  | |  | |  |
| ≤44 | 568 (21.0) | |  | | 117 (16.7) | |  |
| 45 a 64 | 1,542 (57.0) | |  | | 396 (56.6 ) | |  |
| ≥65 | 586 (22.0)) | |  | | 187 (26.7) | |  |
| **Color/race** |  | |  | |  | |  |
| Indigenous/Yellow | 127 (4.7) | |  | | 33 (4.7) | |  |
| White | 1,023 (37.8) | |  | | 265 (37.7) | |  |
| Brown | 1,236 (45.7) | |  | | 322 (45.8) | |  |
| Black | 320 (11.8) | |  | | 83 (11.8) | |  |
| **Marital status** |  | |  | |  | |  |
| Single | 608 (22.5) | |  | | 132 (18.8) | |  |
| married | 1,463 (54.1) | |  | | 393 (55.9) | |  |
| Divorced/Widower | 635 (23.4) | |  | | 178 (25.3) | |  |
| **Retired** |  | |  | |  | |  |
| Yes | 945 (34.9) | |  | | 289 (41.1) | |  |
| No | 1,761 (65.1) | |  | | 414 (58.9) | |  |
| **Family Income**  **(minimum wage)** |  | |  | |  | |  |
| ≤1 | 1,504 (55.6) | |  | | 389 (55.3) | |  |
| >1 | 1,202 (44.4) | |  | | 314 (44.7) | |  |
| **Participates in the "Bolsa Família"**  **Program^c^** |  | |  | |  | |  |
| yes | 485 (18.0) | |  | | 117 (16.7) | |  |
| No | 2,198 (82.0)) | |  | | 583 (83.3) | |  |
| **Education** |  | |  | |  | |  |
| Up to high school, incomplete. | 1,817 (67.2) | |  | | 508 (72.3) | |  |
| High school complete | 889 (32.8) | |  | | 195 (27.7) | |  |
| **Living in the municipality** |  | |  | |  | |  |
| yes | 2,522 (93.2) | |  | | 669 (95.2) | |  |
| No | 184 (6.8) | |  | | 34 (4.8) | |  |
| **Housing Location** |  | |  | |  | |  |
| Urban | 2,297 (85.4) | |  | | 593 (85.0) | |  |
| Rural | 394 (14.6) | |  | | 105 (15.0) | |  |
| **FHS Coverage ^c^** |  | |  | |  | |  |
| Yes | 2,216 (83.6) | |  | | 593 (86.1) | |  |
| No | 436 (16.4) | |  | | 96 (13.9) | |  |
|  |  | |  | |  | |  |
| **Time until the DSC** |  | |  | |  | |  |
| Up to 20 minutes | 1,637 (60.5) | |  | | 422 (60.0) | |  |
| More than 20 minutes | 1,069 (39.5) | |  | | 281 (40.0) | |  |
| **Hours of operation meet the needs** |  | |  | |  | |  |
| Yes | 89 (3.3) | |  | | 22 (3.1) | |  |
| No | 2.617 (96.7) | |  | | 681 (96.9) | |  |
| **Welcoming** |  | |  | |  | |  |
| Good | 2,630 (97.2) | |  | | 685 (97.4) | |  |
| Bad | 13 (0.5) | |  | | 4 (0.6) | |  |
| Fair / Don't know / Did not answer / | 63 (2.3) | |  | | 14 (2.0) | |  |
| **DSC in good conditions** |  | |  | |  | |  |
| Yes | 2,331 (86.1) | |  | | 603 (85.8) | |  |
| No | 375 (13.9) | |  | | 100 (14.2) | |  |
| **DSC service** |  | |  | |  | |  |
| Good | 2,628 (97.1) | |  | | 687 (97.7) | |  |
| Bad | 7 (0.3) | |  | | 2 (0.3) | |  |
| Fair / Don't know / Did not answer / | 71 (2.6) | |  | | 14 (2.0) | |  |

National Program for Improving Access and Quality of Dental Specialty Centers (PMAQ-CEO)

^a^ Prevalence in the column.

^b^ Percent of patients in each category who underwent the prosthesis in a DSC.

^c^ Absolute frequency totals differ due to missing data (blank or ignored data).

**Table 2.** Frequency distribution of sample users according to contextual variables, PMAQ-CEO, Brazil, 2018.

| **Variables** | **Users**  **n (%)** | **n (%)^b^ of patients**  **that made prosthesis in DSC (n= 703)** |
| --- | --- | --- |
| **Region** |  |  |
| Midwest | 182 (9.6) | 49 (7.0) |
| North | 87 (4.6) | 37 (5.3) |
| Northeast | 715 (37.7) | 265 (37.7) |
| South | 221 (11.7) | 86 (12.2) |
| Southeast | 690 (36.4) | 266 (37.8) |
| **DSC Location** |  |  |
| Capital | 406 (15.0) | 82 (11.7) |
| Countryside | 2,300 (85.0) | 621 (88.3) |
| **Makes molding** |  |  |
| Yes | 1,949 (72.0) | 639 (90.9) |
| No | 757 (28.0) | 64 (9.1) |
| **Manages waiting lists** |  |  |
| Yes | 2,030 (75.0) | 548 (78.0) |
| No | 676 (25.0) | 155 (22.0) |
| **There are predefined quotas** |  |  |
| Yes | 621 (23.0) | 206 (29.3) |
| No | 1,370 (50.6) | 433 (61.6) |
| There is no service in this specialty | 715 (26.4) | 64 (9.1) |
| **Waiting time for service at the DSC^s^** |  |  |
| ≤ 2 months | 1,259 (57.9) | 369 (56.2) |
| >2 months | 915 (42.1) | 287 (43.8) |
| **Number of people on the waiting**  **list** |  |  |
| ≤123^d^ | 744 (46.7) | 265 (49.2) |
| >123 | 850 (53.3) | 274 (50.8) |
| **Suspended services due to lack of**  **Supplies / instruments** |  |  |
| Yes | 589 (21.8) | 141 (20.1) |
| No | 2,117 (78.2) | 562 (79.9) |
| **Average delivery of the prosthesis**  **a month** |  |  |
| ≤25 | 820 (30.3) | 212 (30.2) |
| >25 | 1,885 (69.7) | 491 (69.8) |

National Program for Improving Access and Quality of Dental Specialty Centers (PMAQ-CEO) ^a^Prevalence in the column; ^b^Prevalence of patients in each category who underwent prosthesis in a DSC. ^c^Absolute frequency totals differ due to missing data (blank or ignored data). ^d^Median of the sample.

**Table 3.** Crude and adjusted analysis between “having had the prosthesis done at a DSC” according to contextual and individual variables, PMAQ-CEO, Brazil, 2018. (n=2706)

| **Variables** | **OR (CI95%)^a^** | **p-value^b^** | **OR adjusted (CI95%)^a^** | **p-value^b^** |
| --- | --- | --- | --- | --- |
| **Contextual level (DSC)** |  |  |  |  |
| Macro region CO (Ref=No ) | 1.00 (0.52;1.91) | 0.993 |  |  |
| Macro region NE (Ref=No ) | 1.09 (0.61;1.94) | 0.770 |  |  |
| Macro region S (Ref=No ) | 1.04 (0.55;1.98) | 0.896 |  |  |
| Macro region SE (Ref=No ) | 1.26 (0.71;2.25) | 0.433 |  |  |
| Place of DSC (Ref=Capital ) | 1.46 (1.04;2.05) | 0.028 | 1.41 (1.01;1.97) | 0.041 |
| Make impressions (Ref=No) | 1.66 (3.74;7.45) | <0.001 |  |  |
| Management waiting list (Ref=No) | 1.24 (0.98;1.62) | 0.106 |  |  |
| Predefined places (Ref=No) | 1.07 (0.84;1.38) | 0.573 |  |  |
| Time of waiting (Ref>2 months ) | 0.91 (0.72;1.14) | 0.403 |  |  |
| People on the waiting list (Ref>123 ) | 1.16 (0.90;1.49) | 0.236 |  |  |
| Suspended calls (Ref=Yes ) | 1.15 (0.88;1.51) | 0.317 |  |  |
| Number of prostheses per month (Ref≤25 ) | 1.01 (0.79;1.29) | 0.935 |  |  |
| **Individual level** |  |  |  |  |
| Sex (Ref=Male) | 1.12 (0.93;1.35) | 0.240 |  |  |
| Age 45 to 64 years (Ref≤44) | 1.33 (1.03;1.72) | 0.027 |  |  |
| Age ≥65 years (Ref≤44) | 1.81 (1.34;2.43) | <0.001 |  |  |
| Race/color (Ref=White) | 1.00 (0.74;1.35) | 0.990 |  |  |
| Race/color Brown (Ref=White) | 1.00 (0.82;1.23) | 0.941 |  |  |
| Marital marriage (Ref= Single) | 1.32 (1.05;1.68) | 0.019 |  |  |
| Marital widower (Ref= Single) | 1.40 (1.08;1.82) | 0.010 |  |  |
| Retired (Ref=No) | 1.43 (1.20;1.72) | <0.001 |  |  |
| Family income (Ref>1MW ) | 0.99 (0.81;1.20) | 0.893 |  |  |
| “*Bolsa família*” (Ref=No) | 0.88 (0.70;1.11) | 0.291 |  |  |
| Education (Ref≥ High School ) | 1.38 (1.14;1.68) | 0.001 | 1.23 (1.01;1.50) | 0.041 |
| Live in the city (Ref=No) | 1.59 (1.04;2.45) | 0.034 | 1.69 (1.07;2.66) | 0.024 |
| Place of residence (Ref=Urban) | 1.04 (0.82;1.33) | 0.729 |  |  |
| Coverage by FHS (Ref=No) | 1.29 (1.01;1.66) | 0.042 |  |  |
| Time to DSC (Ref>20min ) | 0.97 (0.81;1.17) | 0.774 |  |  |
| Opening hour met the needs (Ref=No ) | 0.93 (0.56;1.55) | 0.789 |  |  |
| Welcoming (Ref=Bad ) | 0.79 (0.22;2.89) | 0.724 |  |  |
| Installations in good condition (Ref= No) | 0.96 (0.74;1.25) | 0.761 |  |  |
| DSC service (Ref=Bad ) | 0.88 (0.16;5.00) | 0.8899 |  |  |

National Program for Improving Access and Quality of Dental Specialty Centers (PMAQ-CEO). ^a^OR: Crude Odds Ratio. P-value: obtained by the multilevel logistic regression analysis. The variance between DSC=0.1390; Residual variance=0.8673; Quasi-likelihood under the Independence model Criterion (empty model)=2407.42.
